# A quasi-experimental evaluation of an interpersonal communication intervention to increase insecticide-treated net use among children in Zambia

**DOI:** 10.1186/1475-2875-11-313

**Published:** 2012-09-07

**Authors:** Joseph Keating, Paul Hutchinson, John M Miller, Adam Bennett, David A Larsen, Busiku Hamainza, Cynthia Changufu, Nicholas Shiliya, Thomas P Eisele

**Affiliations:** 1 Department of Global Health Systems and Development, Tulane University School of Public Health and Tropical Medicine, 1440 Canal Street, Suite 2200, New Orleans, LA 70112, USA; 2 PATH Malaria Control and Evaluation Partnership in Africa (MACEPA), National Malaria Control Centre, Chainama Hospital College Grounds, Great East Road, Lusaka, Zambia; 3 National Malaria Control Centre, Ministry of Health Zambia, Chainama Hospital College Grounds, Great East Road, Lusaka, Zambia; 4 Society for Family Health, Plot 549 Ituna Road, PO Box 50770, Ridgeway, Lusaka, Zambia

**Keywords:** Evaluation, Insecticide-treated net (ITN), Interpersonal communication campaign (IPC), Community health worker (CHW), Malaria, Zambia

## Abstract

**Background:**

This paper presents results from an evaluation of the effect of a community health worker (CHW) –based, interpersonal communication campaign (IPC) for increasing insecticide-treated mosquito net (ITN) use among children in Luangwa District, Zambia, an area with near universal coverage of ITNs and moderate to low malaria parasite prevalence.

**Methods:**

A quasi-experimental community randomized control trial was conducted from 2008 to 2010. CHWs were the unit of randomization. Cross-sectional data were collected from houses in both 2008 and 2010 using simple random sampling of a complete household enumeration of the district. A difference-in -differences approach was used to analyse the data.

**Results:**

ITN use among children <5 years old in households with ≥1 ITN increased overall from 54% in 2008 to 81% in 2010 (*χ*^*2*^ = 96.3, p <0.01); however, there was no difference in increase between the treatment and control arms in 2010 (p >0.05). ITN use also increased among children five to 14 years old from 37% in 2008 to 68% in 2010. There was no indication that the CHW-based intervention activities had a significant effect on increasing ITN use in this context, over and above what is already being done to disseminate information on the importance of using an ITN to prevent malaria infection.

**Discussion:**

ITN use increased dramatically in the district between 2008 and 2010. It is likely that IPC activities in general may have contributed to the observed increase in ITN use, as the increased observed in this study was far higher than the increase observed between 2008 and 2010 malaria indicator survey (MIS) estimates. Contamination across control communities, coupled with linear settlement patterns and subsequent behavioural norms related to communication in the area, likely contributed to the observed increase in net use and null effect in this study.

## Background

Zambia has recently scaled up free insecticide-treated mosquito net (ITN) distribution within rural areas with the objective of achieving universal coverage: at least one ITN per sleeping space. While challenges to increasing ITN ownership may diminish as a result of the expansion of large-scale distribution efforts, ITN impact on transmission will be minimized if they are not properly and consistently used, especially among populations vulnerable to increased malaria morbidity and mortality, such as children and pregnant women.

Given the considerable disparity between household ITN possession and use observed [1-3], malaria control programmes across sub-Saharan Africa (SSA) are developing strategies to promote ITN uptake and use. As such, contextually relevant village-based interpersonal communication (IPC) interventions are being developed to increase ITN possession and use. Such strategies have proven effective for modifying behaviour in a number of malaria endemic contexts [4-6], thereby providing empirical justification for the intervention strategy described herein.

**Figure 1:**
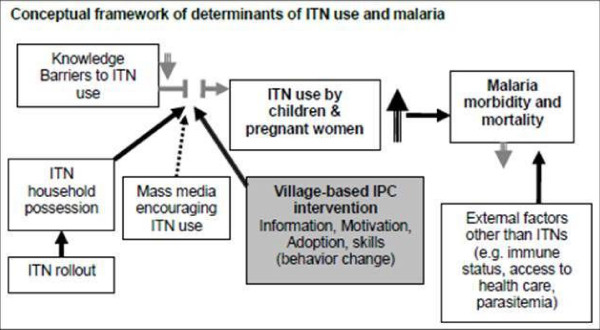
Conceptual framework of the determinants of ITN use.

The IPC intervention in Zambia (Figure 1) was based on the following observations. First, a recent malaria indicator survey (MIS) conducted in Zambia shows that only half (52%) of children used an ITN, demonstrating a clear need to increase ITN use [7]. Second, under trial settings, free ITNs along with demonstrations of proper ITN deployment and use through IPC have been shown to increase ITN coverage and use among children <5 years old [1,8,9]. Third, while there is anecdotal evidence linking behaviour change communication (BCC) to ITN use, to date there has been no empirical evidence quantifying the impact of a CHW-based IPC intervention on ITN use within the Zambian context.

This paper evaluates the effect of a CHW-based IPC intervention for increasing ITN use among children <5 years old in Luangwa District, Zambia, an area with near universal coverage of ITNs and moderate to low malaria parasite prevalence. The paper also lends insight into whether CHW-based interventions can contribute to malaria elimination efforts, what additional steps may be necessary to further achieve sustainable increases in ITN use, and important lessons related to logistics and human behaviour in the context of community randomized control trials (CRCT). Ethical approval for this study was obtained from the Institutional Review Boards (IRB) of Tulane University, the University of Zambia, and the Program for Appropriate Technology in Health (PATH).

## Methods

### Study site description

Luangwa District has a population of approximately 34,000 people [10], and is situated between the Luangwa and Zambezi rivers. This location makes the district vulnerable to malaria transmission throughout the year. In 2005, the district was chosen for a pilot test to scale up malaria control. With the goal of achieving 100% coverage of household ITN possession, the district had received 9,100 free ITNs from the National Malaria Control Center (NMCC) and the PATH Malaria Control and Evaluation Partnership in Africa (MACEPA) project by February 2006, with an additional 7,000 ITNs distributed later that year. Coverage of households with children possessing at least one ITN was 81% in both 2008 and 2010 [11].

Malaria is a leading public health problem in Luangwa District. Primary malaria control and prevention activities are undertaken by the NMCC, with non-governmental organization (NGO) also contributing to the national malaria control strategy. CHWs, comprised of community volunteers, have the task of providing basic health service in their communities, including the planned roll-out of home-based management of malaria [12]. CHWs are managed by health centre personnel. There is approximately one CHW responsible for the delivery of basic health services per 500 community members. CHWs deliver the IPC intervention described below.

### Intervention

The intervention was a community-based IPC intervention rolled out by CHWs over an 18-month period. CHWs were trained and supported as needed with additional resources by the implementing partner in Zambia, MACEPA, in conjunction with the Zambian Ministry of Health (MOH) and the NMCC. This intervention was based on an information, motivation, and behaviour skills model [13]; the intervention was adapted to reflect the current situation in Zambia in the context of known barriers to ITN possession [14]. Although CHWs were not actively encouraging individuals to obtain nets, information on where to obtain nets was given when requested.

The intervention focused on providing accurate information on malaria transmission, malaria prevention through the use of ITNs, net repair and retreatment, who should be using the ITNs, and the importance of using ITNs throughout the year. The information was delivered in a combination of ways. House-to-house visits and visual aids were used to show mothers and heads of households who should be sleeping under the ITNs; pictures and printed leaflets demonstrating how malaria is transmitted were given to households to keep. Community plays and demonstrations were used to provide accurate information on malaria transmission, proper ITN deployment, net repair and retreatment. This intervention information was disseminated four times per year.

A second focus was on providing information for motivating individuals to use ITNs, such as readily understandable statistics on the efficacy and effectiveness of ITNs on reducing malaria morbidity and mortality, the potential benefits of ITNs at reducing nuisance mosquitoes and thereby enhancing sleep, and the cost savings to the family through avoiding malaria infections. This information was delivered in a combination of ways, including house-to-house visits to communicate directly with mothers and heads of households, as well as within community plays and demonstrations. This motivational information was also disseminated four times per year throughout intervention communities.

The last focus was on providing on-site assistance with net deployment, provision of any necessary materials such as string, nails and hammer, and repairing of ITNs. These activities were conducted prior to each rainy season with the trained CHW going house-to-house.

The intervention was piloted within five villages for a one-month period prior to implementation. In-depth interviews were used to collect formative data about the intervention during the pilot phase of the project, which was then used to modify the intervention before implementation to ensure that all activities are culturally and contextually relevant. In addition, results from the baseline survey were used to revise and add to the IPC intervention materials prior to implementation.

### Evaluation study design

**Figure 2:**
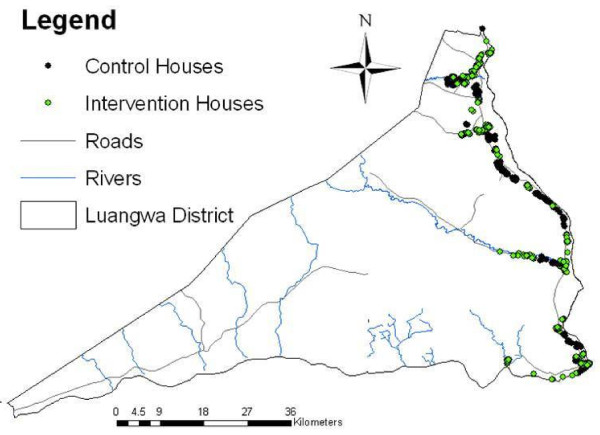
Map of study area.

**Figure 3:**
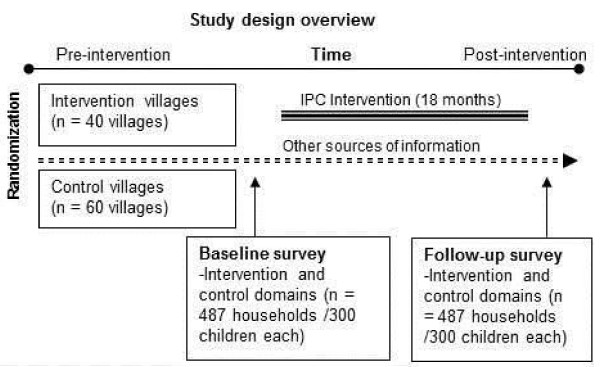
Study design overview.

A two-arm, quasi-experimental, community, randomized, controlled trial pre-post design, was used to quantify the effectiveness of a CHW-based IPC intervention for increasing ITN use among children<5 years old within households possessing at least one ITN, as compared to children within households possessing at least one ITN within control communities receiving no CHW-based IPC intervention, other than exposure to mass media, as is often rolled out throughout Zambia. Figure 2 illustrates the geographic distribution of houses falling into either the treatment or control group. Effectiveness was measured as a double-difference between treatment and control groups over time. The unit of randomization was the CHW catchment area. Random sampling was used to assign 60 CHWs to the treatment group and 40 community health workers to serve as the control group (Note: an error in communication and implementation led to 10 additional CHWs receiving the control group training; as the target was 50/50, this resulted in an uneven distribution of CHWs between treatment and control groups. In the interest of minimizing additional bias, CHWs were not re-randomized, as trainings had already started – this error rendered the design quasi-experimental). The treatment group communities were exposed to the IPC intervention described below by their CHW, while the control group CHWs exposed their community to an HIV awareness campaign that had previously been developed by the Society for Family Health. This was done to limit bias associated with including some CHWs and not others in the malaria-related intervention; moreover, an HIV awareness campaign was chosen to serve as the control because there is no evidence to suggest that exposure to this information changes health behaviour related to malaria. Figure 3 illustrates a conceptual framework for the evaluation design.

### Data collection and study outcomes

A simple random sample of households was drawn in 2008 and 2010 across both the intervention and control group after a complete enumeration of households was conducted. All cross-sectional data were collected during the peak malaria transmission seasons (April to May) for each collection period. A modified MIS-style questionnaire was administered to heads of households, as well as mothers and caregivers of children at randomly selected houses in both the control and intervention communities before (2008) and after (2010) the intervention. Trained data collectors were responsible for implementing the survey. The survey methods have been described elsewhere [11,15] and followed the protocol used by the Zambia National MIS [16]. A total sample size of 1,200 children across both surveys was sought to allow the detection of a 10% increase in ITN use from a baseline of 50% with 80% statistical power, assuming a design effect of 1.35, and the probability of committing a type-1 error set at 5% (1-tailed test). Approximately 800 houses in each survey round were targeted to reach the estimated sample size of children, as not all houses approached contained eligible children.

Household registries and net rosters were used to generate variables related to ITN use. Data collected from mothers and heads of household were used to generate control variables related to household wealth, geography, sex, mother’s education, and age of children <5 years old. ITNs were defined as any net that was treated at least once in previous 12 months, or was a permanently treated net, according to the identified brand and based on WHO recommendations for long-lasting insecticidal nets [17]. The primary outcome was the proportion of children under five years old reporting to have slept under an ITN the night before the survey, within households owning at least one ITN. The proportion of children <5 years old living in households where any ITN was used the night before the survey was used as a secondary outcome. Parasite infection and febrile illness data were also collected from all household residents greater than one month old; these results are presented elsewhere [11,15] and are not the focus of this evaluation.

### Data analysis

All analyses were done using Stata 10.1 (Stata Corporation, College Station, Texas, USA). Chi-square statistics (*χ*^2^) were used to test for differences in ITN use among children <5 years old in treatment *vs* control houses. Chi-square statistics were also used to test for differences in ITN use by geography, sex, wealth, age, and self-reported exposure to the intervention. Wealth quintiles were created using a principle components analysis [18]. A geography variable was created based on distance of house to the Boma, and dichotomized as equal to one if North Luangwa (≥50 km from the Boma) and zero if South Luangwa (<50 km from the Boma). This variable served as a proxy for access to other health-related information, health care, and transmission differences noted in earlier studies [14,15]. The age variable was categorized as zero to 11 months old, 12–23 months old, 24–35 months old, 36–47 months old, and 48–59 months old. Mother’s education was dichotomized as having attended primary grade 6 or higher or primary grade 5 and below.

Logistic regressions were used to test whether the CHW-based IPC intervention increased ITN use among children<5 years old, while controlling for age, sex, wealth, and geography at the individual level using a difference-in-differences approach (i.e., time – intervention interaction term); and secondly, whether the CHW-based IPC intervention increased ITN use among children<5 years old sleeping in a house where any ITN was used, while controlling for the same variables.

An additional analysis was done with the same outcomes, whereby self-reported exposure to the intervention (irrespective of intervention *vs* control group assignation) was substituted with the intervention variable; self-exposure to the intervention was defined as equal to one if child lived in a house where a CHW either disseminated information or assisted with hanging up the net and equal to zero if not. For this analysis, two models were run. One using only 2010 (post-intervention) data, as no CHW-based IPC intervention was in place during the 2008 baseline survey, and one using both 2008 and 2010 data to account for any messaging or IPC interventions reported.

For all analyses, the probability of committing a type-1 error (alpha) was set at 0.05. Standard errors were adjusted to account for correlated data at the household level. All bivariate and regression results involving ITN use as the outcome are among children living in ITN-owning houses. Eisele and colleagues [11] report on additional ITN related analyses.

## Results

A total of 1,595 houses were selected for the surveys, of which 914 contained children <5 (N = 483 in 2008 and N = 431 in 2010), yielding a sample of 1,402 children with complete data (N = 737 in 2008 and N = 667 in 2010). The distribution of children living in houses located in treatment *vs* control communities was similar on sex, age, distance to Boma, and mother’s education (p >0.05) at baseline (Table 1). Household wealth quintiles were different between intervention and control communities at baseline; 25.0% of children in the intervention communities resided in houses reporting high wealth, *vs* 11.3% in control communities (*χ*^*2*^ = 38.3, p<0.01). The mean number of ITNs per house was the same between intervention and control communities at baseline (2.0) and at follow-up (2.1); no statistically significant difference was detected (p > 0.05). ITN use was also higher in intervention groups at baseline (60.8% in intervention communities *vs* 41% in control communities), rendering this design quasi-experimental (*χ*^*2*^ = 21.2, p<0.01).

**Table 1:** **Descriptive statistics for children under five years old in control*vs*intervention communities in sampled houses at baseline (2008)**

	**Intervention (*n = 469*)**		**Control (*n = 266*)**	
	**%**	**95% CI**	**%**	**95% CI**
**Age in years**				
0-11 months	22.2	18.4-25.9	24.8	19.6-30.0
12-23 months	19.8	16.2-23.5	18.8	14.1-23.5
24-35 months	19.6	16.0-23.2	16.5	12.1-21.0
36-47 months	17.9	14.4-21.4	19.6	14.8-24.3
48-59 months	20.5	16.8-24.1	20.3	15.5-25.2
**Sex**				
Male	50.1	45.6-54.6	47.4	41.4-53.4
Female	49.9	45.4-54.4	52.6	46.6-58.7
**Geography**				
Southern Luangwa (<50 km from Boma)	45.8	41.3-50.4	45.9	39.9-51.9
Northern Luangwa (≥50 km from Boma)	54.2	49.6-58.7	54.1	48.1-60.1
**Mother’s education**				
None – Primary 6	47.8	43.2-52.3	49.3	43.3-55.3
Primary 7 - Higher	52.2	47.7-56.8	50.8	44.7-65.8
**Household wealth**				
Poorest	18.8	15.2-22.3	22.2	17.2-27.2
Poor	23.0	19.2-26.7	14.7	10.4-18.9
Middle	16.0	12.7-19.3	27.4	22.1-32.8
Rich	17.3	13.8-20.7	24.4	19.3-29.6
Richest	25.0**	21.0-28.9	11.3	7.5-15.1
**ITN use**				
ITN used last night by child^§^	60.8**	55.9-65.6	41.0	34.2-47.7
Any ITN use in house last night^§^	61.3**	56.4-66.1	43.4	37.1-50.7

* *P* <0.05; ** *P* <0.01; § Among children living in ITN-owning houses.

### IPC intervention exposure

At the post-intervention survey, about half of children were located in intervention communities (47.5%) and half in control communities (52.5%). Only 47.0% of children in the intervention communities were located in houses where respondents reported exposure to the CHW-based IPC intervention; conversely, 30.6% of children in control houses reported exposure to the intervention.

At follow-up in 2010, 90.2% (95% CI: 87.7–92.7) of children lived in houses where respondents reported that they had heard malaria information within the previous 12 months from any source. Exposure to the three intervention components break down as follows: 27.2% (95% CI: 23.4–30.9) of children lived in houses where the respondent reported that they had heard malaria information from a CHW (intervention-1) during the intervention period (12 months); 27.1% (95% CI: 23.3–30.8) of children were located in houses that reported that a CHW assisted with hanging a net (intervention-2), while 37.7% (33.6–41.8) of children were located in houses where respondents reported that malaria information was disseminated to the house (intervention-3).

**Figure 4:**
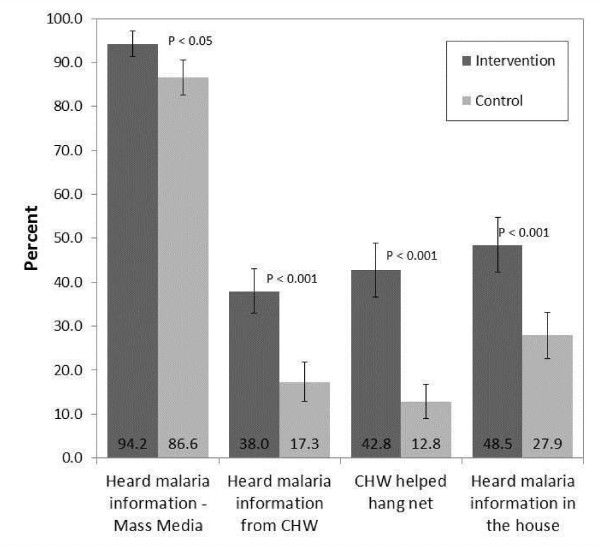
Exposure to IPC interventions and malaria messaging at time of follow-up survey (2010).

Figure 4 illustrates the percent of children in houses exposed to the respective intervention components in treatment *vs* control communities at the post-intervention survey. A significantly higher proportion of exposure was observed in intervention communities, suggesting overall the intervention rolled out more in intervention communities, compared to control communities. An analysis of baseline values in relation to treatment versus control shows that fewer than 15% of houses were exposed to any one of the three interventions, with no statistical differences between children in intervention *vs* control communities detected (p >0.05). These results suggest that exposure to CHW-based IPC interventions prior to the intervention roll-out was minimal.

### Insecticide-treated net use

Results from the baseline survey in 2008 show that 81.0% of children were in houses that possessed at least one ITN (95% CI: 78.2–83.8) and that 81.1% (95% CI: 78.1–84.1) of households in the study area possessed ≥1 ITN at follow-up in 2010 [19].

**Figure 5:**
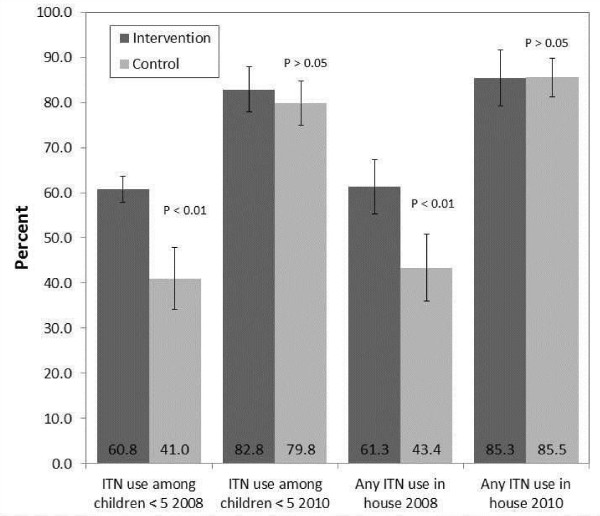
ITN use among children in ITN-owning households.

Figure 5 shows ITN use among children in ITN-owning households between intervention and control communities. In 2008 ITN use was low within ITN-owning households located in control communities, with just under half [41.0% (95% CI: 34.2–47.8)] of children sleeping under an ITN the night before the survey. Among children in ITN-owning households located in treatment communities, ITNs were used by 60.8% (95% CI: 55.9–65.6%) of children the night before the survey (*χ*^*2*^ = 20.2, p <0.01). Overall, ITN use significantly increased among children<5 years old from 54% at baseline in 2008 to 81% at follow-up (*χ*^*2*^ = 96.3, p <0.01) in 2010, irrespective of treatment group assignation. Similar relationships were found with the outcome of any ITN use in the house. ITN use also increased among children five to 14 years old from 37.3% (95% CI: 34.4-40.3) in 2008 to 67.5% (95% CI: 64.4-70.4) in 2010.

Using a per-protocol analysis of self-reported exposure (did the respondent report that the household was exposed to either malaria messages in the house, CHW assisted hanging of an ITN, or hearing malaria messages from a CHW) irrespective of treatment *vs* control group membership, no differences were observed between self-reported exposure and the outcomes of ITN use among children and any ITN use in the house in at the time of the follow-up survey (2010).

### Multivariate regression analyses

Results from two logistic regressions, controlling for child age, sex, distance to the Boma, wealth, mother’s education, time and treatment *vs* control in the study area suggest that neither using an ITN the night before the survey nor the use of any ITN within the house the night before the survey were significantly related to the CHW-based intervention. The time by treatment group interaction analyses (i.e., difference-in-differences analyses) failed to show significant effects (Table 2). The main effects models do however show significant positive relationships between child use of an ITN and being in the intervention group (O.R. 2.24 95% CI: 1.46–3.43) and the year 2010 (O.R. 5.76 95% CI: 3.45–9.62) separately, as well as between any ITN use in the house and being in the intervention group (O.R. 2.01, 95% CI: 1.35–3.13) and the year 2010 (O.R. 7.88, 95% CI: 4.72–13.2) separately. No control variables tested significant in these analyses.

**Table 2:** Logistic regressions predicting odds of ITN use among children under five years old living in ITN-owning houses in Luangwa District, Zambia

	**Child used an ITN the night before survey (*n = 1,136*)**		**Any ITN use in house the night before the survey (*n = 1,136*)**	
	**O.R.**	**95% CI**	**O.R.**	**95% CI**
**Intervention Community**	2.24**	1.46-3.43	2.06**	1.35-3.13
**Time (2010)**	5.76**	3.45-9.62	7.88**	4.72-13.17
Time*intervention	0.55	0.28-1.09	0.47	0.24-0.96
**Age in years**				
0-11 months	Ref		Ref	
12-23 months	2.00	0.73-1.65	1.04	0.68-1.59
24-35 months	1.12	0.81-1.76	1.10	0.73-1.66
36-47 months	1.03	0.69-1.54	0.88	0.58-1.34
48-59 months	0.78	0.53-1.15	0.74	0.50-1.12
**Sex**				
Male	Ref		Ref	
Female	1.04	0.81-1.36	1.01	0.77-1.33
**Geography**				
Southern Luangwa (<50 km from Boma)	Ref		Ref	
Northern Luangwa (≥50 km from Boma)	1.01	0.73-1.40	1.07	0.77-1.50
**Mother’s education**				
None – Primary 6	Ref		Ref	
Primary 7 and Higher	0.85	0.62-1.18	0.77	0.55-1.08
**Household wealth**				
Poorest	Ref		Ref	
Poor	0.93	0.56-1.56	0.73	0.43-1.23
Middle	1.09	0.64-1.84	0.99	0.58-1.69
Rich	1.07	0.63-1.83	1.05	0.61-1.80
Richest	1.12	0.67-1.86	1.05	0.62-1.79
Pseudo R^2^	8.9%		11.1%	

* *P* <0.05; ** *P* <0.01

The results of the additional regressions performed using a self-reported exposure variable (e.g., was the house exposed to CHW-based activities) and year 2010, in lieu of intervention *vs* control group assignation were similar to the results presented above, and also show that neither CHW-based activities nor disseminated information at the household level to be related to increases in ITN use in this context. However, when using data from both 2008 and 2010, self-reported exposure to CHW-based activities (e.g., CHW assisted hanging of net, CHW disseminated information, or hearing malaria information in the house) was associated with almost a two-fold increase in the odds of a child using an ITN the previous night (O.R. 1.74, 95% CI: 1.23–2.46).

## Discussion

ITN use among children dramatically increased from 54% in 2008 to 81% in 2010, irrespective of treatment *vs* control group membership within this context, while the quasi-experimental evaluation showed no significant effect of CHW-based malaria IPC activities on ITN use among children over this time. However, the increases observed were much greater than the increases observed using Zambia MIS estimates over this same time period. Rural Zambia reported <5 ITN use at 48.6% (95% CI: 43.6–53.6) in 2008 and 65.7% (95% CI: 61.8–68.1) in 2010, much lower than observed in Luangwa District. As well, there were associations observed between ITN use and self-reported exposure to any malaria messages in the community, strengthening the argument that information dissemination does contribute in some way. While these results provide no definitive evidence of an effect, they do provide important lessons and observations that may help explain these null results.

There is some evidence to suggest that this intervention was rolled-out across both arms of the study, thus exposure within the control group communities likely occurred. Three forces are suspected to have been in play to influence this unanticipated exposure. First, settlement patterns in Luangwa District are linear, in that most houses are located close to the main road traversing the District in a general north–south direction (Figure 2). Linear settlement patterns often facilitate interaction and communication as a function of access to roadways (per obs) or the Boma (note: the Boma is the economic and governmental hub of the District). Second, many communities in this District are similar in cultural, educational, and economic behaviour; it is possible that these similarities create opportunities for increased communication at the individual level within the communities. Lastly, programme records suggest that some CHWs assigned to the control group also disseminated malaria-related information. While this likely exposed control group houses to the intervention, many CHWs felt compelled to increase malaria awareness via information dissemination. These observations are supported by the results showing many houses in control group communities reported receiving CHW-based malaria activities, either assistance with hanging a net or information dissemination.

A likely bi-product of possible contamination was that ITN use significantly increased across both arms between 2008 and 2010. While the intervention group had higher ITN use at baseline, skewing the results from the onset, the control group showed an increase in ITN use by almost two-fold, and the intervention group close to 25%. While there is no empirical evidence demonstrating a relationship between the IPC intervention and the outcomes, it is clear that some form of communication is working, as evidenced by large proportions of respondents in both arms of the study reported hearing any malaria message from any source. Further, the results show that ITN use by a child is very similar to any ITN use in the house, suggesting that in houses where nets were being used, the child was under the net. The results also suggest that ITNs are not being used for purposes other than the intended use; also a key message of the IPC intervention. These observations may or may not be the result of the IPC intervention, as many information sources report on the importance of children sleeping under ITNs to protect against malaria infection, as well as on the importance of proper use in general.

The pooled, cross-sectional data analysis showed associations between self-reported exposure to malaria messages and interactions with CHWs and the outcomes of ITN use. While these results support frameworks suggesting linkages between malaria messaging and behavioural change (Figure 1), much of the data driving the significant relationships were reported prior to the CHW-based intervention activities (i.e., baseline data) and across both arms (i.e., messaging from any source was already high in the study area). However, these results are subject to endogeneity and selection bias, and could also be the result of exposure to other malaria messaging systems prior to 2008. As such, this increase may not represent a true increase across the study area as a result of the CHW-based intervention in this study. Preliminary analysis showed that both mother’s education and distance to the Boma were related to self-reported exposure; this suggests that those with high education obtain information from multiple sources, increasing the likelihood of intervention exposure, and those close to the Boma are at a higher likelihood of exposure from any source, given that the Boma likely has many more media and social outlets than nearby rural areas.

## Conclusions

In conclusion, the data provide no definitive evidence that CHW-based activities increase ITN use in the context of near universal coverage of ITNs and moderate malaria transmission. However, there is evidence to suggest that malaria messaging in general is an important tool for increasing awareness. Given the observations related to contamination, linear settlement patterns and strong communication ties, community-level IPC interventions may play an important role for influencing health behaviour change.

## Abbreviations

CHW, Community Health Worker; ITN, Insecticide-Treated bed Net; IPC, Interpersonal Communication; BCC, Behaviour Change Communication; CRCT, Community Randomized Control trials; PATH, Program for Appropriate Technology in Health; IRB, Institutional Review Board; NMCC, National Malaria Control Center; MACEPA, Malaria Control and Evaluation Partnership in Africa; NGO, Non-governmental organizations; MOH, Zambia Ministry of Health; MIS, Malaria Indicator Survey.

## Competing interests

The authors declare that they have no competing interests.

## Authors’ contributions

JK led the analysis and drafting of the paper; DAL assisted with the study roll-out and reviewed all drafts; PH assisted with the editing and review; JM, AB and TPE assisted with the drafting and editing of the paper; BH, CC and NS led the study roll-out, provided manuscript edits and review. All authors read and approved the final manuscript.
